# Impact of Inhaled Corticosteroids on Osteoporosis in Chronic Obstructive Pulmonary Disease (COPD): A Systematic Review

**DOI:** 10.7759/cureus.89201

**Published:** 2025-08-01

**Authors:** Mona Mohammednoor, Ahmed Mohamed Elamin Mubarak Osman, Saba Hamad, Fatima Ismail Abdelhalim Ismail, Mohammed Elamien Yousif Elsharief Mohammed Elamien, Esra Eltahir Mohamed Gamareldin, Md Hasanur Rahman

**Affiliations:** 1 Diabetes and Endocrinology, Queen Elizabeth Hospital Birmingham, Birmingham, GBR; 2 General Medicine, Jouf University Medical Services Center, Sakaka, SAU; 3 Gastroenterology, Henry Ford Health System, Detroit, USA; 4 Pulmonology, Dr. Muhammad Alfagih Hospital, Riyadh, SAU; 5 Family Medicine, Riyadh Second Health Cluster, Riyadh, SAU; 6 Faculty of Medicine, Assiut University, Assiut, EGY; 7 General Medicine, Luton & Dunstable University Hospital, Luton, GBR

**Keywords:** bone health, copd, fracture risk, inhaled corticosteroids, osteoporosis, systematic review

## Abstract

Inhaled corticosteroids (ICS) are commonly prescribed for chronic obstructive pulmonary disease (COPD) management, but their long-term use has been associated with potential adverse effects on bone health, including osteoporosis and fractures. This systematic review aimed to evaluate the impact of ICS on osteoporosis and fracture risk in COPD patients by synthesizing evidence from observational and clinical studies. A comprehensive literature search was conducted across PubMed, Web of Science, Scopus, Embase, and the Cochrane Library, following Preferred Reporting Items for Systematic Reviews and Meta-Analyses (PRISMA) guidelines. Studies were screened for eligibility based on predefined inclusion criteria, and data were extracted using a standardized form. The Newcastle-Ottawa Scale (NOS) was employed to assess the risk of bias in included studies. Due to heterogeneity in study designs and outcome measures, a narrative synthesis was performed, focusing on key themes such as dose-response relationships, fracture risk, and the influence of ICS duration. Eleven studies were included, revealing mixed findings on the association between ICS use and bone health outcomes. While some studies reported a dose-dependent increase in osteoporosis incidence and fracture risk, others found no significant association or even protective effects in specific subgroups. High-dose and long-term ICS use were consistently linked to greater risks, particularly in vulnerable populations such as elderly and female patients. The methodological quality of studies varied, with most demonstrating low to moderate risk of bias. This review highlights the complex relationship between ICS use and bone health in COPD patients, emphasizing the importance of individualized treatment approaches. While ICS remain essential for COPD management, clinicians should consider bone-protective strategies in high-risk patients, particularly those on long-term or high-dose regimens. Future research should standardize exposure and outcome definitions to facilitate more robust quantitative synthesis.

## Introduction and background

Chronic obstructive pulmonary disease (COPD) remains a leading cause of morbidity and mortality worldwide, with an estimated global prevalence of over 300 million individuals [1,2]. Characterized by persistent respiratory symptoms and airflow limitation, COPD imposes a significant healthcare burden due to its progressive nature and associated systemic complications. Inhaled corticosteroids (ICS) have been an integral component of pharmacological management for COPD, particularly in patients with frequent exacerbations and severe airflow limitation [3]. By reducing airway inflammation and exacerbation frequency, ICS use has contributed to improved disease control and patient quality of life [4].

However, the systemic effects of ICS, particularly on bone health, have raised concerns among clinicians and researchers. Osteoporosis is a prevalent comorbidity in COPD patients, attributed to factors such as chronic inflammation, reduced physical activity, hypogonadism, nutritional deficiencies, and the frequent use of systemic corticosteroids [5]. ICS, despite being primarily delivered to the airways, undergo a degree of systemic absorption, which may potentially influence bone metabolism and accelerate bone mineral density (BMD) loss. The magnitude of this risk, however, remains uncertain due to inconsistent findings across studies and variability in ICS formulations, dosages, and durations of use [6].

Previous studies investigating the association between ICS use and osteoporosis risk in COPD patients have yielded conflicting results [7-9]. While some observational studies have reported significant reductions in BMD and increased fracture risk among ICS users, others have found minimal or no clinically relevant effects. In addition, discrepancies exist in study designs, outcome measures, and adjustment for confounding factors such as smoking status, body mass index, and baseline bone health, further complicating the interpretation of available evidence.

Given the clinical importance of optimizing COPD treatment while minimizing adverse outcomes, a comprehensive evaluation of the existing literature is warranted to clarify the relationship between ICS use and osteoporosis in this population [3-5]. Such insights are critical for guiding risk-benefit decisions in pharmacotherapy and informing preventive strategies to mitigate bone health deterioration in COPD patients.

In this systematic review, we aim to synthesize the current evidence on the impact of inhaled corticosteroids on osteoporosis and bone mineral density among patients with COPD. We will critically appraise and narratively summarize findings from observational and interventional studies to better inform clinicians and researchers about the potential skeletal risks associated with ICS use in this vulnerable population.

## Review

Methodology

Eligibility Criteria

This systematic review was conducted in accordance with the Preferred Reporting Items for Systematic Reviews and Meta-Analyses (PRISMA) 2020 guidelines [10]. Studies were considered eligible if they investigated the impact of ICS on osteoporosis or BMD among patients with COPD. Randomized controlled trials, cohort studies, and case-control studies published in peer-reviewed journals were included. Exclusion criteria comprised editorials, letters, conference abstracts, reviews, and studies lacking relevant outcomes.

Information Sources

The literature search included the following electronic databases: PubMed, Web of Science, Scopus, Embase, and the Cochrane Library. All databases were searched from their inception dates until June 2025 to ensure comprehensive coverage of the available literature.

Search Strategy

A systematic search strategy was developed using Medical Subject Headings (MeSH) terms and relevant keywords related to "COPD", "inhaled corticosteroids", and "osteoporosis". The full search strategy for PubMed is presented in Table 1, and it was adapted appropriately for the syntax and indexing of other databases. The detailed search strategy is also attached in the Appendix. No language restrictions were applied. To enhance completeness, reference lists of all included studies and relevant reviews were also manually screened to identify additional eligible studies.

**Table 1:** PubMed search strategy COPD: chronic obstructive pulmonary disease, ICS: inhaled corticosteroids, BMD: bone mineral density

Search number	Search query
1	“Pulmonary Disease, Chronic Obstructive”[Mesh] OR COPD OR “chronic obstructive pulmonary disease”
2	“Adrenal Cortex Hormones”[Mesh] OR “inhaled corticosteroids” OR “ICS” OR budesonide OR fluticasone OR beclomethasone OR mometasone OR ciclesonide
3	“Osteoporosis”[Mesh] OR osteoporosis OR “bone mineral density” OR BMD OR “bone loss”
4	1 AND 2 AND 3

Selection Process

All identified citations were imported into EndNote X9 (Clarivate Analytics, Philadelphia, PA, USA) for systematic management, and duplicates were removed using the software’s automated duplicate detection function, followed by manual verification. Two reviewers independently screened titles and abstracts for relevance, and potentially eligible studies were retrieved in full text for detailed assessment. Discrepancies were resolved through discussion or adjudication by a third reviewer to ensure consistency and minimize selection bias.

Data Collection Process

Data extraction was performed independently by two reviewers using a standardized and pilot-tested data extraction form. Extracted data included study characteristics (first author, year of publication, country, and study design), population characteristics (sample size, mean age, and gender distribution), details of ICS exposure (type, dose, and duration), comparator groups, outcome measures (such as BMD changes or osteoporosis incidence), follow-up duration, and key findings. Disagreements in data extraction were resolved by consensus.

Data Items

The data items extracted focused on the PICOS framework: participants, interventions, comparators, outcomes, and study designs. Specific attention was paid to adjustment for potential confounders in individual studies.

Study Risk-of-Bias Assessment

The risk of bias in observational studies was assessed independently by two reviewers using the Newcastle-Ottawa Scale (NOS) [11], which evaluates study quality based on participant selection, comparability of groups, and outcome assessment. Any disagreements were resolved through discussion to reach a final consensus rating for each included study.

Effect Measures

Due to the heterogeneity in reporting formats, outcome units, and statistical adjustments across studies, no single effect measure was pooled. Instead, original reported effect sizes (e.g., odds ratios, hazard ratios, and mean differences) were extracted for narrative synthesis.

Synthesis Methods

Meta-analysis or other statistical pooling was not performed due to substantial clinical and methodological heterogeneity among the included studies. This heterogeneity was observed across several dimensions. First, studies used different types of inhaled corticosteroids (e.g., fluticasone, budesonide, and beclomethasone) administered in varying doses and treatment durations, which could influence their impact on bone metabolism differently. Second, the methods and anatomical sites for assessing BMD varied, with some studies using dual-energy X-ray absorptiometry (DXA) at the lumbar spine, while others assessed the femoral neck or total hip, affecting comparability of outcomes. Third, definitions of osteoporosis were inconsistent; some studies applied World Health Organization T-score criteria, while others used varying thresholds or diagnostic criteria. Lastly, the extent of adjustment for confounding factors such as age, sex, smoking status, physical activity, and baseline BMD differed significantly across studies, introducing further methodological inconsistency.

Pooling such diverse data statistically could lead to biased or misleading conclusions due to non-comparability and potential effect modification. Therefore, a narrative synthesis was undertaken to systematically summarize and interpret the findings while respecting the heterogeneity in study designs, populations, interventions, and outcomes.

Results

Study Selection

**Figure 1:**
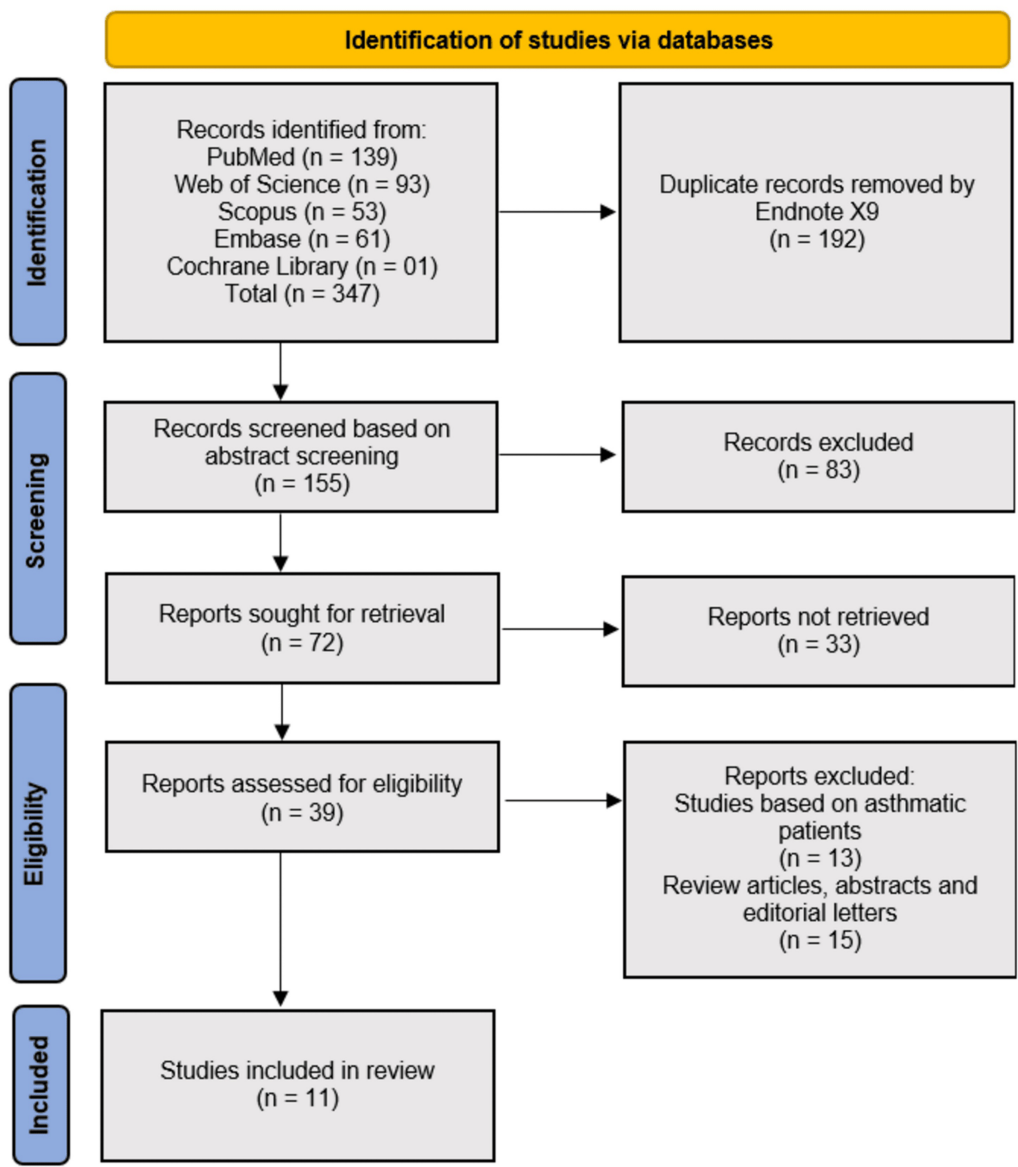
PRISMA flowchart detailing literature search and study selection methodology PRISMA: Preferred Reporting Items for Systematic Reviews and Meta-Analyses

The initial search across multiple databases, including PubMed, Web of Science, Scopus, Embase, and the Cochrane Library, yielded a total of 347 records. After removing 192 duplicate records using EndNote X9, 155 studies remained for abstract screening. Of these, 83 records were excluded as they did not meet the inclusion criteria. The remaining 72 full-text reports were sought for retrieval, of which 33 could not be obtained. A total of 39 full-text articles were assessed for eligibility. Among these, 13 studies were excluded for focusing on asthmatic patients, and 15 were excluded as they were review articles, abstracts, or editorial letters. Ultimately, 11 studies [12-22] met the inclusion criteria and were included in the systematic review (Figure 1).

Overview of the Included Studies

The systematic review included 11 studies [12-22] investigating the impact of ICS on osteoporosis and fracture risk in patients with COPD. The studies were conducted across diverse geographical regions, including the UK, Taiwan, the USA, Sweden, Canada, and China, and employed various study designs such as cohort studies, nested case-control studies, and cross-sectional analyses (Table 2). Sample sizes ranged from 85 participants in a prospective cross-sectional study [22] to 318,385 individuals in a retrospective cohort study [21]. The populations primarily comprised COPD patients aged 40 years or older, with some studies focusing on specific subgroups, such as females [13] or veterans [16].

**Table 2:** Key characteristics and findings from the included studies ICS: inhaled corticosteroids, LABD: long-acting bronchodilator, COPD: chronic obstructive pulmonary disease, FEV1: forced expiratory volume in one second, FSC: fluticasone propionate/salmeterol fixed-dose combination, QCT: quantitative computed tomography, BMI: body mass index, BMD: bone mineral density, FVC: forced vital capacity, NR: not reported, OR: odds ratio, UK: United Kingdom, USA: United States of America

Author (year)	Location	Study design	Sample size (n)	Population characteristics	Intervention (ICS type and dose)	Comparator	Outcome measures	Follow-up duration	Key findings
Price et al., [12] (2019)	UK	Matched cohort study using two large databases	Diabetes onset: 17,970; Diabetes progression: 804; Osteoporosis onset: 19,898	Patients ≥40 years old with COPD initiating ICS or LABD from 1990–2015	ICS, mean exposure ≥500 µg/day (fluticasone propionate–equivalent)	LABD	Diabetes onset, diabetes progression, osteoporosis onset	Median 3.7–5.6 years per treatment group	ICS vs LABD significantly increased risk of diabetes onset. No overall increase in diabetes progression or osteoporosis onset. However, at ≥500 µg/day ICS exposure, risk was significantly increased for all three outcomes, showing dose–response relationships.
Liu et al., [13] (2016)	Taiwan	Retrospective cohort, population-based study	10,723 (ICS users: 812; nonusers: 9,911)	Newly diagnosed female COPD patients ≥40 years old, no prior osteoporosis or asthma diagnosis	ICS (type not specified), dose categories: ≤20 mg, >20–60 mg, >60 mg	Non-ICS users	Incidence of osteoporosis	1997–2011 (up to ~14 years follow-up)	ICS use associated with reduced osteoporosis incidence; dose–response protective effect observed.
Miller et al., [14] (2010)	UK	Nested case-control study within a population-based cohort	53,191 (1,523 fracture cases)	Patients aged ≥45 years with COPD	Fluticasone propionate/salmeterol fixed-dose combination (FSC); doses classified as low, medium, high, or very high (fluticasone propionate equivalents)	Other ICS use or no ICS use	Incident nonvertebral fractures	2003–2006 (exposure assessed in the year prior to index date)	FSC use in the year prior associated with increased odds of nonvertebral fractures; no increased odds with other ICS use; no dose-response relationship observed; ever use of FSC slightly elevated fracture risk.
Johannes et al., [15] (2005)	USA	Nested case-control within a cohort	89,877 cohort; 1,722 cases; 17,220 controls	Adults ≥40 years with COPD or asthma enrolled in UnitedHealthcare from 1997-2001	Various ICS (including fluticasone propionate); dose not specified in abstract (estimated cumulative dose over 0–6m, 7–12m, 0–12m assessed)	No ICS exposure	First treated nonvertebral fracture	Up to 12 months exposure assessment prior to index date	No increased nonvertebral fracture risk with ICS exposure overall or with fluticasone propionate; no dose-response effect; in COPD-only subgroup, recent ICS exposure not associated with increased fracture risk.
Lee and Weiss, [16] (2004)	USA	Nested case–control study	1,708 cases with nonvertebral fractures matched to 6,817 controls (Total cohort: 40,157 COPD patients)	94% male; mean age 62.7 years; Veterans Affairs patients with COPD	ICS use (dose converted to beclomethasone equivalents); current high-dose ICS users ≥700 μg/day	Patients with no ICS exposure	Nonvertebral fracture risk	Follow-up occurred during observation period post-1999 diagnosis	Overall ICS use not associated with increased fracture risk. Current high-dose ICS use associated with increased fracture risk. Female COPD patients using ICS showed dose–response protective effect for osteoporosis.
Pujades-Rodriguez et al., [17] (2007)	UK	Nested case-control study	Cases: 1235; Controls: 4598	COPD patients aged ≥40 years; mean FEV1 ~57.5% (cases) and 58.5% (controls)	Inhaled corticosteroids (type not specified); median dose cases: 269 mcg/day, controls: 226 mcg/day; effect assessed up to ≥1600 mcg/day	No ICS use or lower ICS doses	Fracture risk	Data from 1 Jan 1998 – 5 July 2005	Fracture risk increased with higher ICS doses; OR for ≥1600 mcg/day was 1.80. Effect persisted after adjusting for FEV1 and oral corticosteroid use.
Chiu et al., [18] (2021)	Taiwan	Nested case–control study (population-based)	232,192 (58,048 cases; 174,144 controls)	COPD patients retrieved from Taiwan National Health Insurance Research Database (2002–2017)	ICS use (type and dose not specified in abstract)	Non-ICS users / matched controls	Osteoporosis or osteoporotic fractures (claims-based diagnosis)	3 years before index date	ICS use associated with a 1.053-fold increased osteoporosis risk; New ICS use linked to increased osteoporosis risk regardless of exposure period; Female patients showed dose–response protective effect.
Janson et al., [19] (2021)	Sweden	Observational cohort study using electronic medical record data linked to National Health Registries	9651 COPD patients and 59,454 matched reference controls	Patients with COPD from 52 Swedish primary care centres (2000-2014)	ICS (type not specified); stratified by dose (low-dose vs. high-dose)	COPD patients not using ICS and matched reference controls	Any osteoporosis-related event (all fractures, fractures typically related to osteoporosis, recorded osteoporosis diagnosis, prescriptions of osteoporosis drugs, combined osteoporosis-related event)	Data from 2000-2014)	COPD patients had higher risk of osteoporosis-related events vs controls. Dose–effect relationship: High-dose ICS significantly increased risk of osteoporosis-related events, low-dose ICS also increased risk compared to COPD patients not using ICS.
Gonzalez et al., [20] (2018)	Canada (Quebec)	Nested case-control analysis within a cohort study	240,110	COPD patients (men and women; cohort from 1990-2005, mean age not stated)	ICS use; fluticasone equivalents ≥1000 μg daily for >4 years	No ICS use	Hip or upper extremity fractures	Mean 5.3 years	Any ICS use not associated with fracture risk; however, >4 years of high-dose ICS use (≥1000 μg) modestly increased fracture risk with no difference between sexes.
Pace et al., [21] (2025)	USA	Retrospective cohort study using electronic health record data (prevalent and inception cohorts)	Prevalent cohort: 318,385; Inception cohort: 209,062	Individuals aged >45 years with COPD	Long-term ICS use (>24 months)	Short-term ICS use (<4 months)	Composite outcome: new diagnosis of type 2 diabetes, cataracts, pneumonia, osteoporosis, or nontraumatic fracture; recurrent pneumonia; recurrent nontraumatic fracture	Comparison of >24 months vs <4 months ICS use implies minimum 24 months follow-up for long-term group	Long-term ICS use was associated with significantly greater rates of composite outcomes in both cohorts, increased recurrent pneumonia, and increased recurrent fractures.
Gao et al., [22] (2025)	China (Gansu Provincial People's Hospital)	Prospective cross-sectional study	85	COPD patients; data on age, sex, BMI, smoking status, tea-drinking habits, physical activity	NR (ICS type and dose not reported)	NR	BMD measured via QCT; lung function (FEV1/FVC%)	NR	Significant positive relationship between BMD and FEV1/FVC% overall. Non-linear analysis showed BMD positively correlated with FEV1/FVC% before breakpoint (128.08 mg/cm³), but negative and non-significant after. Highlights need for individualized COPD management focusing on bone health.

Association Between ICS Use and Osteoporosis

The findings on the association between ICS use and osteoporosis were mixed. Liu et al. [13] reported a protective effect of ICS use in female COPD patients, with a dose-response relationship showing reduced osteoporosis incidence at higher doses (HR: 0.73, 95% CI: 0.63-0.84). Conversely, Chiu et al. [18] found a slight but significant increase in osteoporosis risk with ICS use (OR = 1.053, 95% CI: 1.020-1.087). Similarly, Janson et al. [19] observed a dose-dependent increase in osteoporosis-related events, with high-dose ICS associated with a higher risk (RR: 1.52, 95% CI: 1.24-1.62) compared to low-dose ICS (RR: 1.27, 95% CI: 1.13-1.56). Price et al. [12] noted no overall increase in osteoporosis risk with ICS use but identified a significant dose-response effect at doses ≥500 µg/day.

ICS Use and Fracture Risk

The relationship between ICS use and fracture risk also varied across studies. Miller et al. [14] reported increased odds of nonvertebral fractures with fluticasone propionate/salmeterol fixed-dose combination use (OR: 1.25, 95% CI: 1.07-1.47), while Johannes et al. [15] found no significant increase in fracture risk with ICS exposure in COPD patients (OR: 0.86, 95% CI: 0.59-1.25). High-dose ICS (≥700 µg/day) was associated with elevated fracture risk in some studies (Lee and Weiss, 2004; OR: 1.68, 95% CI: 1.10-2.57), and Pujades-Rodriguez et al. [17] reported a similar trend for doses ≥1600 mcg/day (OR: 1.74, 95% CI: 1.00-3.01). Gonzalez et al. [20] found that prolonged high-dose ICS use (>4 years at ≥1000 µg/day) modestly increased fracture risk (OR: 1.10, 95% CI: 1.02-1.19).

Dose-Response Relationships and Confounding Factors

Several studies highlighted dose-response relationships, with higher ICS doses consistently linked to greater risks of osteoporosis or fractures (Table 3). For instance, Pace et al. [21] reported a significantly increased risk of composite outcomes, including osteoporosis, with long-term ICS use (HR: 2.60, 95% CI: 2.56-2.64). Adjustments for confounders such as age, sex, comorbidities, and oral corticosteroid use were common across studies, but residual confounding remained a limitation in some analyses [12,18].

**Table 3:** Summary of statistical findings from included studies CI: confidence interval, HR: hazard ratio, ICS: inhaled corticosteroids, FSC: fluticasone/salmeterol combination, COPD: chronic obstructive pulmonary disease, OR: odds ratio, FEV1: forced expiratory volume in one second, FVC: forced vital capacity, RR: relative risk, BMD: bone mineral density, BMI: body mass index, NR: not reported, β: beta coefficient

Author (year)	Outcome assessed	Effect size	95% CI	p-value	Direction of effect	Adjustment for confounders
Price et al., [12] (2019)	Osteoporosis onset	1.13	0.93–1.39	NR	No significant effect overall; increased risk at ≥500 µg/day (dose-response)	Adjusted for residual confounding after matching
Liu et al., [13] (2016)	Incidence of osteoporosis	HR: 0.73 (overall); Dose-response HRs: ≤20 mg: 0.84, >20–≤60 mg: 0.78, >60 mg: 0.72	Overall: 0.63–0.84; ≤20 mg: 0.69–1.04; >20–≤60 mg: 0.59–1.04; >60 mg: 0.55–0.96	Overall p<0.001; p for trend=0.0023	Reduced risk with ICS use, stronger effect at higher doses	Adjusted for age, income, and medications
Miller et al., [14] (2010)	Nonvertebral fracture (FSC use vs. no use)	1.25	1.07–1.47	<0.05	Increased odds with FSC use	Adjusted using conditional logistic regression for potential confounders
Johannes et al., [15] (2005)	Nonvertebral fracture risk (any ICS exposure within 30 days in COPD patients)	0.86	0.59 – 1.25	NR	No significant increase in risk	Adjusted for demographics, oral corticosteroid and other medication exposure, comorbidities, indicators of respiratory disease severity
Lee and Weiss, [16] (2004)	Nonvertebral fractures (current high-dose ICS ⩾700 μg/day vs. no exposure)	1.68	1.10–2.57	NR	Increased risk with high-dose ICS use	Adjusted for confounders in conditional logistic regression models
Pujades-Rodriguez et al., [17] (2007)	Fracture risk	OR 1.74 (highest dose exposure ≥1600 mcg/day)	1.00–3.01	p for trend 0.007	Increased fracture risk with higher ICS dose	Adjusted for mean % predicted FEV1 and annual prescription rate for oral corticosteroids
Chiu et al., [18] (2021)	Osteoporosis risk associated with ICS use in COPD	OR = 1.053	1.020–1.087	NR	Increased risk	Adjusted for potential confounders
Janson et al., [19] (2021)	Any osteoporosis-related event (including all fractures, osteoporotic fractures, osteoporosis diagnosis, and prescriptions)	High-dose ICS: RR 1.52; Low-dose ICS: RR 1.27	High-dose: 1.24–1.62; Low-dose: 1.13–1.56	<0.0001	Increased risk with dose–effect relationship	Multivariate analysis (adjusted for confounders)
Gonzalez et al., [20] (2018)	Hip or upper extremity fractures with >4 years ICS use at daily doses ≥1000 μg fluticasone equivalents	1.10	1.02–1.19	NR	Increased risk	Adjusted for age, sex, follow-up time, dose, duration; conditional logistic regression
Pace et al., [21] (2025)	Composite outcome (including osteoporosis) (Inception cohort)	2.60	2.56 – 2.64	<0.001	Increased risk with long-term ICS use	Adjusted (electronic health record-based hazard ratio analysis)
Gao et al., [22] (2025)	Relationship between BMD and FEV1/FVC%	β = 0.1 (overall); before breakpoint β = 0.245; after breakpoint β = -0.136	Overall: 0.1–0.1	Overall: <0.0001; before breakpoint: 0.0019; after breakpoint: 0.0753	Positive overall; Positive before breakpoint; Negative (non-significant) after breakpoint	Adjusted for age, sex, BMI, smoking status, tea-drinking habits, and physical activity

Heterogeneity in Findings

The heterogeneity in findings may be attributed to differences in study populations, ICS types and doses, follow-up durations, and outcome definitions. For example, Gao et al. [22] explored the relationship between BMD and lung function, revealing a non-linear association that further complicates the interpretation of ICS effects on bone health in COPD patients.

Risk-of-Bias Results

The risk-of-bias assessment using the NOS indicated that most included studies were of low risk. Specifically, Price et al. [12], Liu et al. [13], Lee and Weiss [16], Pujades-Rodriguez et al. [17], Chiu et al. [18], Janson et al. [19], Gonzalez et al. [20], and Pace et al. [21] scored between 7 and 9, reflecting a low risk of bias. Two studies, Miller et al. [14] and Johannes et al. [15], had moderate risk of bias with total scores of 6 due to lower comparability ratings. Only one study, Gao et al. [22], was assessed as having high risk of bias with a score of 5, primarily due to lower selection and comparability scores (Table 4). Overall, the methodological quality of the included studies was acceptable, with the majority demonstrating low risk of bias.

**Table 4:** Risk-of-bias results

Author (year)	Study design	Selection (Max 4)	Comparability (Max 2)	Outcome/exposure (Max 3)	Total score (Max 9)	Risk of bias
Price et al., [12] (2019)	Matched cohort study	4	2	3	9	Low
Liu et al., [13] (2016)	Retrospective cohort	3	2	2	7	Low
Miller et al., [14] (2010)	Nested case control	3	1	2	6	Moderate
Johannes et al., [15] (2005)	Nested case control	3	1	2	6	Moderate
Lee and Weiss, [16] (2004)	Nested case control	3	2	2	7	Low
Pujades-Rodriguez et al., [17] (2007)	Nested case control	3	2	2	7	Low
Chiu et al., [18] (2021)	Nested case control	4	2	3	9	Low
Janson et al., [19] (2021)	Observational cohort	4	2	3	9	Low
Gonzalez et al., [20] (2018)	Nested case control	3	2	2	7	Low
Pace et al., [21] (2025)	Retrospective cohort	4	2	3	9	Low
Gao et al., [22] (2025)	Cross-sectional	2	1	2	5	High

Discussion

The findings of this systematic review highlight the complex relationship between ICS use and osteoporosis or fracture risk in patients with COPD. The included studies, encompassing diverse populations and methodologies, present a nuanced picture of this association, with results varying by dose, duration of exposure, and patient characteristics. The evidence suggests that while ICS use may not universally increase the risk of osteoporosis or fractures, higher doses and prolonged exposure are consistently associated with greater risks, particularly in vulnerable subgroups. This discussion synthesizes these findings, compares them with existing literature, and explores their clinical implications.

One of the most striking observations from this review is the dose-dependent relationship between ICS use and adverse bone outcomes. Studies such as Price et al. [12] and Janson et al. [19] demonstrated that higher ICS doses (e.g., ≥500 µg/day fluticasone propionate-equivalent) were linked to increased risks of osteoporosis onset and fracture, respectively. This aligns with previous research indicating that systemic absorption of ICS, even at low levels, can suppress bone formation and accelerate bone loss over time [23]. The dose-response gradient observed in our review supports the hypothesis that cumulative ICS exposure is a critical determinant of bone health deterioration in COPD patients. Notably, Pace et al. [21] further reinforced this by showing that long-term ICS use (>24 months) significantly elevated the risk of composite outcomes, including osteoporosis and fractures. These findings are consistent with meta-analyses by Shang et al. [24], which reported a 1.5-fold increased fracture risk with high-dose ICS in COPD populations.

However, the relationship between ICS and bone health is not uniformly detrimental. Liu et al. [13] reported a paradoxical protective effect of ICS on osteoporosis incidence in female COPD patients, particularly at higher doses. This contrasts with the majority of studies in this review and may reflect gender-specific differences in bone metabolism or unmeasured confounding factors, such as vitamin D status or physical activity levels. Similarly, Lee and Weiss [16] found that female COPD patients using ICS exhibited a dose-response protective effect against osteoporosis, suggesting potential hormonal interactions or selection biases in these subgroups. Such discrepancies underscore the need for sex-stratified analyses in future research, as women with COPD are already at higher baseline risk for osteoporosis due to postmenopausal bone loss [25].

The variability in fracture risk across studies further complicates the interpretation of ICS-related bone effects. While Miller et al. [14] and Pujades-Rodriguez et al. [17] reported elevated fracture risks with specific ICS formulations or high-dose exposure, Johannes et al. [15] found no significant association. These inconsistencies may stem from differences in study design, outcome definitions (e.g., vertebral vs. nonvertebral fractures), or adjustment for confounders like oral corticosteroid use, which independently exacerbates bone loss [26]. For instance, Gonzalez et al. [20] adjusted for oral corticosteroid exposure and still identified a modest increase in fracture risk with prolonged high-dose ICS use, suggesting that ICS alone may contribute to bone fragility. This is corroborated by mechanistic studies showing that ICS reduce osteoblast activity and intestinal calcium absorption, even in the absence of systemic corticosteroid co-administration [27].

The role of underlying COPD severity as a confounder cannot be overlooked. Several studies in this review, including Janson et al. [19] and Chiu et al. [18], adjusted for markers of respiratory disease severity (e.g., FEV1%), yet residual confounding likely persists. COPD itself is associated with systemic inflammation, reduced physical activity, and malnutrition-all of which contribute to osteoporosis [28]. Thus, disentangling the direct effects of ICS from the sequelae of advanced COPD remains challenging. Gao et al. [22] attempted to address this by examining the relationship between BMD and lung function, revealing a non-linear association that complicates causal inference. Their findings suggest that COPD-related factors may dominate bone health outcomes at severe disease stages, potentially masking ICS effects.

Geographic and healthcare system differences may also explain some heterogeneity. For example, Liu et al. [13] and Chiu et al. [18], both conducted in Taiwan, reported divergent results despite similar populations. Variations in ICS prescribing practices, genetic susceptibility to osteoporosis, or access to bone health monitoring (e.g., DEXA scans) could underlie these disparities. In contrast, studies from the UK and Sweden [12, 19] demonstrated more consistent dose-response relationships, possibly reflecting standardized ICS regimens and robust electronic health record systems for outcome ascertainment.

Clinically, these findings emphasize the need for individualized ICS prescribing in COPD. While ICS remain a cornerstone of COPD management for reducing exacerbations [29], their bone-related risks necessitate careful risk-benefit evaluation, especially in high-dose or long-term users. Current guidelines recommend periodic BMD assessments for COPD patients on ICS [30], yet adherence to these recommendations is often suboptimal [31]. Our review supports the prioritization of non-ICS therapies (e.g., long-acting bronchodilators) in patients with pre-existing osteoporosis or high fracture risk, as suggested by Price et al. [12]. For those requiring ICS, concomitant bone-protective measures-such as calcium/vitamin D supplementation or bisphosphonates-should be considered, particularly in postmenopausal women or elderly patients [32].

Limitations

This review has several limitations. First, the predominance of observational studies precludes causal inference, and residual confounding (e.g., smoking, physical activity) may bias the results. Second, heterogeneity in ICS types, doses, and outcome definitions limits direct comparability across studies. Third, the exclusion of non-English studies and reliance on published data may introduce selection bias. Finally, the cross-sectional design of Gao et al. [22] and small sample size weaken the generalizability of their findings.

## Conclusions

ICS use in COPD is associated with dose-dependent risks of osteoporosis and fractures, although the magnitude of risk varies by population and ICS regimen. Clinicians must weigh these risks against the benefits of ICS in reducing COPD exacerbations, particularly in high-risk subgroups. Future research should prioritize prospective studies with standardized bone outcomes and rigorous adjustment for COPD severity to clarify these associations. Until then, vigilant monitoring and preventive strategies are essential to mitigate ICS-related bone harm in this vulnerable population.

## Appendices

**Table 5:** Detailed search strategy for all databases

Database	Search strategy
PubMed	("Pulmonary Disease, Chronic Obstructive"[Mesh] OR "COPD" OR "chronic obstructive pulmonary disease") AND ("Adrenal Cortex Hormones"[Mesh] OR "Inhaled corticosteroids" OR "ICS" OR "fluticasone" OR "budesonide" OR "beclomethasone") AND ("Osteoporosis"[Mesh] OR "Bone Mineral Density"[Mesh] OR "osteopenia" OR "bone loss" OR "fracture risk")
Web of Science	TS=("chronic obstructive pulmonary disease" OR "COPD") AND TS=("inhaled corticosteroids" OR "ICS" OR "fluticasone" OR "budesonide" OR "beclomethasone") AND TS=("osteoporosis" OR "bone mineral density" OR "bone loss" OR "fracture risk")
Scopus	TITLE-ABS-KEY("chronic obstructive pulmonary disease" OR "COPD") AND TITLE-ABS-KEY("inhaled corticosteroids" OR "ICS" OR "fluticasone" OR "budesonide" OR "beclomethasone") AND TITLE-ABS-KEY("osteoporosis" OR "bone mineral density" OR "bone loss" OR "fracture risk")
Embase	'chronic obstructive lung disease'/exp OR COPD OR 'chronic obstructive pulmonary disease' AND 'inhaled corticosteroid'/exp OR 'inhaled corticosteroids' OR ICS OR fluticasone OR budesonide OR beclomethasone AND 'osteoporosis'/exp OR 'bone mineral density'/exp OR osteopenia OR 'bone loss' OR 'fracture risk'
Cochrane Library	("chronic obstructive pulmonary disease" OR COPD):ti,ab,kw AND ("inhaled corticosteroids" OR ICS OR fluticasone OR budesonide OR beclomethasone):ti,ab,kw AND ("osteoporosis" OR "bone mineral density" OR "bone loss" OR "fracture risk"):ti,ab,kw
